# A preliminary study on the reference intervals of serum tumor marker in apparently healthy elderly population in southwestern China using real-world data

**DOI:** 10.1186/s12885-024-12408-1

**Published:** 2024-05-29

**Authors:** Qiang Miao, Shuting Lei, Fengyu Chen, Qian Niu, Han Luo, Bei Cai

**Affiliations:** 1 Department of Laboratory Medicine, West China Hospital, Sichuan University, Chengdu, Sichuan China; 2 Division of Thyroid and Parathyroid Surgery, West China Hospital, Sichuan University, Chengdu, Sichuan China; 3 Sichuan Clinical Research Center for Laboratory Medicine, Chengdu, Sichuan China; 4 Clinical Laboratory Medicine Research Center of West China Hospital, No.37, Guoxue Xiang, Wuhou District, Chengdu, Sichuan 610041 China

**Keywords:** Geriatric laboratory medicine, Reference interval, Tumor marker, Real-world data, Indirect method

## Abstract

**Background:**

The aim is to establish and verify reference intervals (RIs) for serum tumor markers for an apparently healthy elderly population in Southwestern China using an indirect method.

**Methods:**

Data from 35,635 apparently healthy elderly individuals aged 60 years and above were obtained in West China Hospital from April 2020 to December 2021. We utilized the Box-Cox conversion combined with the Tukey method to normalize the data and eliminate outliers. Subgroups are divided according to gender and age to examine the division of RIs. The Z-test was used to compare differences between groups, and 95% distribution RIs were calculated using a nonparametric method.

**Results:**

In the study, we observed that the RIs for serum ferritin and Des-γ-carboxy prothrombin (DCP) were wider for men, ranging from 64.18 to 865.80 ng/ml and 14.00 to 33.00 mAU/ml, respectively, compared to women, whose ranges were 52.58 to 585.88 ng/ml and 13.00 to 29.00 mAU/ml. For other biomarkers, the overall RIs were established as follows: alpha-fetoprotein (AFP) 0–6.75 ng/ml, carcinoembryonic antigen (CEA) 0–4.85 ng/ml, carbohydrate antigen15-3 (CA15-3) for females 0–22.00 U/ml, carbohydrate antigen19-9 (CA19-9) 0–28.10 U/ml, carbohydrate antigen125 (CA125) 0–20.96 U/ml, cytokeratin 19 fragment (CYFRA21-1) 0–4.66 U/ml, neuron-specific enolase (NSE) 0–19.41 ng/ml, total and free prostate-specific antigens (tPSA and fPSA) for males 0–5.26 ng/ml and 0–1.09 ng/ml. The RIs for all these biomarkers have been validated through our rigorous processes.

**Conclusion:**

This study preliminarily established 95% RIs for an apparently healthy elderly population in Southwestern China. Using real-world data and an indirect method, simple and reliable RIs for an elderly population can be both established and verified, which are suitable for application in various clinical laboratories.

**Supplementary Information:**

The online version contains supplementary material available at 10.1186/s12885-024-12408-1.

## Background

Malignant tumors comprise of a group of diseases that seriously threaten human health and life [1]. According to estimates from the World Health Organization (WHO) in 2019, malignant tumors were the first or second cause of death among individuals younger than 70 years in 112 countries [2]. Whether in developed or developing countries, the harm caused by malignant tumors cannot be ignored [3]. With the aging of the population in China, the incidence of tumors and the mortality associated with them has been increasing. The prevention and control of malignant tumors have become a public health problem that require great attention [4–6]. Tumor markers play an important role in the screening, auxiliary diagnosis, prognosis, treatment, and monitoring of malignant tumors in high-risk populations because their detection is simple, repeatable, and cause little trauma [7].

The reference interval (RI) is crucial for the accurate interpretation of clinical laboratory test results and one of the critical sources that clinicians use to judge whether the individual is healthy and to make clinical decisions [8]. Aging changes tissue morphology, physiological function, and biochemical immunity of various organ systems in elderly individuals. Exploring the physiological indicators of elderly individuals and formulating the RIs according to the factors to be evaluated has important clinical and sociological significance when studying the etiology, pathology, and clinical diagnosis of diseases that commonly and frequently occur in elderly individuals [9]. Recent studies have consistently demonstrated that RIs vary between elderly populations and non-elderly groups; thus, it is essential to establish distinct RIs for the elderly that differ from those of non-elderly individuals [10–12]. Tumor markers, as an important means of early detection of tumors and observation of curative effects, have also been shown to exhibit significant physiological changes with age, such as AFP, CEA, CYFRA21-1, tPSA increase with age [13–15], and CA72-4 decreases with age [16]. However, collecting qualified and sufficient individuals from special groups, such as newborns, children, and elderly individuals, during the process of establishing RIs using a direct method is challenging; therefore, there have been relatively few studies of RIs for laboratory indicators for these special groups. To date, no large-scale, systematic RI studies of multiple serum tumor markers based on age and sex have been carried out for the elderly population. Due to weakened immune systems and other age-related factors, elderly individuals are more susceptible to malignant tumors. The establishment of accurate, appropriate, and personalized tumor marker RIs is crucial for early screening, treatment, and monitoring of malignant tumors in elderly individuals. The formulation of RIs for serum tumor markers in the elderly population is an urgent task for medical laboratories.

In this study, we utilized real-world data collected from the Laboratory Information System (LIS) of the Department of Laboratory Medicine at West China Hospital between April 2020 and December 2021, we employed an indirect method to establish and verify the RIs for serum tumor markers in an apparently healthy elderly population in Southwestern China. Our findings provide a framework for establishing RIs for specific groups and contribute to the development of geriatric laboratory medicine.

## Methods

### Study subjects

From April 2020 to December 2021, we gathered data on apparently healthy individuals aged 60 years and above who underwent physical examinations from the LIS of the Department of Laboratory Medicine at West China Hospital. For those with multiple test records, to ensure the most current health status, we selected the latest results using their ID numbers and exam dates. Exclusion criteria included abnormal levels of serum alanine aminotransferase (> 50 U/L for males, > 40 U/L for females); aspartate aminotransferase (> 40 U/L for males, > 35 U/L for females); serum creatinine (> 111 µmol/L for males, > 81 µmol/L for females); white blood cell count (outside 3.5–9.5 × 10^9^/L); hemoglobin (< 120 g/L for males, < 110 g/L for females); and any history of oncology, recent surgery, or hospitalization. Samples marked with ‘hemolysis, jaundice, or lipid blood’, which could interfere with accurate test results, were also excluded. The final cohort included 35,635 seniors, with 21,814 men and 13,821 women. Their data encompassed serum tumor markers such as AFP, CEA, CA15-3, CA19-9, CA 125, CYFRA21-1, NSE, tPSA, fPSA, ferritin, and DCP. This study protocol was approved by the Ethics Committee of the West China Hospital, Sichuan University (No. 2020 − 823). All methods were performed in accordance with the relevant guidelines and regulations.

### Instruments and reagents

Fasting venous blood (2–4 ml) was collected from all individuals, after which the serum was separated and tested. AFP, CEA, CA15-3, CA19-9, CA125, CYFRA21-1, NSE, tPSA, fPSA, and ferritin were tested using an electrochemiluminescence immunoassay (Cobas e801; Roche) and its accompanying reagents and calibrators. Lyphochek Tumor Marker Plus Control (Bio-Rad) was used as the control material. A Fuji GC1200 chemiluminescence analyzer and matching reagents, control material, and calibrators were used for testing and quality control of DCP. Our laboratory uses Westgard multi-rules (1_3S_, 2_2S_, and R_4S_) for internal quality control. The Cumulative Coefficient of Variation (CV) is a statistical measure used to assess the relative variability of a dataset over a period of time or across different conditions and it can be particularly useful in fields like quality control or laboratory medicine. The CV is calculated as the ratio of the standard deviation to the mean, and it is often expressed as a percentage. When you have multiple CVs from different periods or groups, the cumulative CV would be the overall assessment of variability across all those periods or groups. In this study, the cumulative coefficient of variation for both high and low-level quality controls in the laboratory during April 2020 and December 2021 was under 5%. The laboratory testing program participates annually in external quality assessment activities organized by the National Centre for Clinical Laboratories and the College of American Pathologists consistently achieving satisfactory results.

### Establishment of reference intervals

#### Normality test and transformation

The skewness–kurtosis normality test was used to analyze data normality. Generally, if the absolute value of kurtosis is less than 10 and the absolute value of skewness is less than 3, the distribution can be considered approximately normal [17]. For data that did not conform to a normal distribution, we used R to perform the Box-Cox normality transformation (where λ is the parameter to be determined and is obtained by the maximum likelihood method). The normality of the transformed data was again analyzed using the skewness-kurtosis test, and λ was calculated as follows:

1 $$Y(\lambda ) = \left\{ {\begin{array}{*{20}{c}}{\frac{{{X^\lambda } - {1^{}}}}{\lambda }\,\,\,\lambda \ne 0} \\ {ln\left( X \right)\,\,\,\lambda = 0} \end{array}} \right\}$$

Here, (X) is the original data, and (λ) is the transformation parameter. The value of (λ) is chosen to best normalize the data. For positive values of (X), the Box-Cox transformation can take on many forms: when (λ = 2), it’s a square transformation; when (λ = 0.5), it’s a square root transformation; and when (λ = 0), it’s a ln transformation. To perform a Box-Cox transformation, one typically uses statistical software (like R or Python) that will find the optimal (λ) value that maximizes the likelihood of the transformed data being normally distributed. In this study, after Box-Cox transformation, analyte levels changed from non-normal to approximately normal distribution, as detailed in Table 1.

**Table 1:** The normality test results after the Box-Cox transformation

Analyte	λ	*N*	Mean	SD	Skewness (95%CI)	Kurtosis (95%CI)
AFP(ng/ml)	-0.16	35,457	1.05	0.38	-0.025 (-0.051, 0.001)	0.63 (0.58, 0.68)
CEA(ng/ml)	-0.10	35,443	0.73	0.49	-0.039 (-0.065, -0.013)	1.09 (1.04, 1.14)
CA15-3(U/ml)	-0.08	13,367	2.08	0.41	0.002 (-0.040, 0.044)	-0.23 (-0.31, -0.15)
CA19-9(U/ml)	0.20	31,614	3.00	1.20	0.149 (0.121, 0.177)	3.40 (3.34, 3.46)
CA125(U/ml)	-0.26	11,010	1.75	0.23	-0.017 (-0.064, 0.030)	0.64 (0.55, 0.73)
CYFRA21-1(ng/ml)	-0.21	5437	0.80	0.33	-0.009 (-0.075, 0.057)	0.19 (0.06, 0.32)
NSE(ng/ml)	-0.72	3288	1.17	0.04	-0.070 (-0.155, 0.015)	1.13 (0.96, 1.30)
tPSA(ng/ml)	0.11	21,660	0.33	1.02	0.180 (0.147, 0.213)	2.51 (2.44, 2.58)
fPSA(ng/ml)	0.11	20,363	-0.94	0.72	0.139 (0.105, 0.173)	2.65 (2.58, 2.72)
Ferritin(ng/ml)	0.19	1822	9.72	1.95	0.038 (-0.077, 0.153)	0.28 (0.05, 0.51)
DCP (mAU/ml)	-0.99	4515	0.96	0.01	-0.143 (-0.216, -0.070)	1.41 (1.26, 1.56)

Abbreviations: AFP, alpha-fetoprotein; CEA, carcinoembryonic antigen; CA, carbohydrate antigen; CYFRA21-1, cytokeratin 19 fragment; NSE, neuron-specific enolase; tPSA, total prostate-specific antigen; fPSA, free prostate-specific antigen; DCP, Des-γ-carboxy prothrombin; CI, confidence interval

#### Outlier rejection

Data were normally transformed and outliers were removed using the Tukey method, which is a robust technique for outlier detection. The upper limit was calculated as P_75_ + 1.5 × interquartile range, and the lower limit was calculated as P_25_ − 1.5 × interquartile range [18]. Any data outside this range were deemed outliers. We removed data outside this range and repeated the process until all outliers were removed. The distribution characteristics of the data after outlier removal using the Tukey method are detailed in Table 2.

**Table 2:** The interquartile range of study groups

Analyte	Gender	*N*	P25	P75	IQR	Min	Max
AFP (ng/ml)	Male	19,494	2.36	4.17	1.81	1.07	11.60
	Female	12,851	2.41	4.38	1.97	1.07	11.60
CEA (ng/ml)	Male	19,484	1.63	3.16	1.53	0.59	9.45
	Female	12,847	1.38	2.70	1.32	0.59	9.39
CA15-3 (U/ml)	Female	12,491	6.88	13.80	6.92	2.56	42.90
CA19-9 (U/ml)	Male	17,752	7.27	15.80	8.53	1.51	44.30
	Female	10,955	6.97	15.50	8.54	1.51	44.30
CA125 (U/ml)	Male	5439	7.89	13.80	5.92	3.89	37.90
	Female	4424	7.90	13.40	5.50	3.89	38.00
CYFRA21-1 (ng/ml)	Male	3485	1.90	3.15	1.25	0.93	7.74
	Female	1367	1.76	3.01	1.25	0.93	7.21
NSE (ng/ml)	Male	2018	11.10	14.81	3.71	8.06	26.81
	Female	868	11.70	15.34	3.64	8.03	26.70
tPSA (ng/ml)	Male	19,454	0.73	2.24	1.51	0.09	10.82
fPSA (ng/ml)	Male	18,259	0.23	0.56	0.33	0.05	1.91
Ferritin (ng/ml)	Male	983	181.50	427.7	246.20	30.9	1075.00
	Female	634	130.20	292.00	161.80	35.9	929.00
DCP (mAU/ml)	Male	2318	18.00	24.00	6.00	13.00	46.00
	Female	1725	16.00	21.00	5.00	13.00	48.00

Abbreviations: AFP, alpha-fetoprotein; CEA, carcinoembryonic antigen; CA, carbohydrate antigen; CYFRA21-1, cytokeratin 19 fragment; NSE, neuron-specific enolase; tPSA, total prostate-specific antigen; fPSA, free prostate-specific antigen; DCP, Des-γ-carboxy prothrombin; *P25*, 25th percentile; *P75*, 75th percentile; *IQR*, interquartile range

#### Investigating group factors

Data were categorized by sex and age. First, we created a scatter plot of RIs related to age to to visually assess the overall trend of various indicators in the subjects as they relate to age, and to determine if there is a clear correlation. At the same time, referring to the age division algorithm based on the clinical laboratory database in Peng et al. research, the decision tree method is used to recommend the best split point. The R^2^ is used to evaluate the fit of all subclasses after each stage and each division step, with a value range from 0 (no fit) to 1 (perfect fit). When the R^2^ of a cutoff point is the highest, it is recommended to use the best split point [19, 20]. Then the standard normal deviate test (Z-test) was used as an objective evaluation criterion to assess whether to partition RIs by the subclass. If Z > Z*, then the difference between the two was considered statistically significant and grouping was required to establish the RI [21]. Z was the calculated statistic and Z* was the judgment limit. Z and Z* were calculated as follows:

2 $$Z = \frac{{\left| {\bar X1 - \bar X2} \right|}}{{{{\left[ {\left( {\frac{{S{1^2}}}{{N1}}} \right)+\left( {\frac{{S{2^2}}}{{N2}}} \right)} \right]}^{\frac{1}{2}}}}},\,{Z^*} = 3{\left[ {\frac{{\left( {N1 + N2} \right)}}{{240}}} \right]^{\frac{1}{2}}}\left( {N1 \geqslant 120,{\mkern 1mu} N2 \geqslant 120} \right)$$

where $\stackrel{-}{\text{X}}1$ and $\stackrel{-}{\text{X}}2$ are the means of the two compared groups, S1 and S2 are the standard deviations of the two groups, and *N*1 and *N*2 are the number of reference values in each subclass.

#### Determination of reference intervals

We calculated the reference interval set for the 95% distribution based on the widely-accepted recommendations of CLSI EP28-A3c. The lower reference limit of 2.5% (P2.5) and the upper reference limit of 97.5% (P97.5) of the 95% distribution for each set of data were calculated using the nonparametric method, and the 90% confidence intervals for P2.5 and P97.5 were calculated using bootstrap. The RIs were selected unilaterally or bilaterally according to the actual clinical significance of each indicator, thus establishing tumor marker estimation RIs for the elderly population in Southwestern China. The RIs for ferritin and DCP were bilateral, from P2.5 to P97.5. The RIs for the remaining indicators, which are clinically significant only when they exceed a certain threshold, were established as unilateral upper limits, ranging from 0 to P95.

#### Verification of reference intervals

Validation data for the RIs were obtained from a population that underwent physical examinations between January 2022 and March 2022. When subjects had multiple test records, only the most recent results were selected. A total of 2780 apparently healthy elderly individuals (1492 men and 1288 women) were included following the previously established inclusion and exclusion criteria. We validated the applicability of the newly established RIs for the requirements of CLSI EP28-A3c and the PRC health industry standards WS/T 402–2012 [22]. The amount of data used for validation met the minimum requirement of at least 20 cases for each indicator in each group. If the ratio of individuals outside the RI was less than 10%, this indicated that the RI was statistically robust. Furthermore, the reference intervals provided by manufacturer were also verified using the same data.

### Statistical methods

All data were analyzed by SPSS 23.0 (IBM Inc., Armonk, NY, USA) and R language (version 3.6.3; R Core Team, 2020). Continuous quantitative variables were described as the mean ± standard deviation or percentiles. Continuous data were checked for a normal distribution using the skewness-kurtosis test. The Box-Cox normal transformation of the data was performed using the R language. Z-tests were used to determine whether the RI needed to be classified according to subclasses. Statistical significance was set at *P* < 0.05.

## Results

### Investigation results of grouping factors

Interestingly, statistical analysis has revealed that there is no need to establish separate RIs for CA125 between elderly men and women (Z < Z*). Since CA15-3 data were exclusive to females, and tPSA and fPSA data to males, the sex division of the RI was not assessed. The Z-test results indicate that the other markers AFP, CEA, CA19-9, CYFRA21-1, and NSE also do not require gender-specific RIs (Z < Z*). Only ferritin and DCP required sex-based grouping to establish RIs (Z > Z*, Supplementary Table S1). We further investigated the necessity of partitioning RIs for common tumor markers according to age within the population. Initially, we analyzed the correlation between various indicators and age. The results showed that in the elderly population over 60 years old, except for AFP and ferritin, which are negatively correlated with age, the rest of the indicators are positively correlated. However, all tumor markers have a weak correlation with age and do not show a significant trend as age increases (Figure S1). Then, we applied the decision tree method to analyze the potential best age split points. The results show that the recommended best age split points for CEA and CYFRA21-1 are both 71 years old, tPSA and fPSA are both 70 years old, CA19-9 and male ferritin are 74 and 68 years old respectively. The remaining indicators do not need to be divided by age as there are no recommended best age split points (Supplementary Table S2). Further objective Z-test statistical analysis shows that there is no need to further divide the age subclass reference interval for the above 6 indicators in the elderly population over 60 years old (Supplementary Table S3).

### Establishment of reference intervals

According to the results of grouping factors, the calculated RIs for tumor markers in the elderly population of Southwestern China were established. Notably, ferritin levels in males surpassed those in females, registering at 64.18–865.80 ng/ml and 52.28–585.88 ng/ml, respectively. A similar trend was observed with DCP levels, where males exhibited higher levels (14.00–33.00 mAU/ml) in comparison to females (13.00–29.00 mAU/ml). Across the remaining analytes, sex and age did not significantly influence the results, yielding the following comprehensive RIs: AFP at 0 to 6.75 ng/ml; CEA at 0 to 4.85 ng/ml; CA15-3 (females only) at 0 to 22.00 U/ml; CA19-9 at 0 to 28.10 U/ml; CA125 at 0 to 20.96 U/ml; CYFRA21-1 at 0 to 4.66 ng/ml; NSE at 0 to 19.41 ng/ml; tPSA (males only) at 0 to 5.26 ng/ml; and fPSA (males only) at 0 to 1.09 ng/ml (Table 3).

**Table 3:** The calculated reference intervals of tumor markers for elderly population

Analyte	Gender	Age(years)	*N*	Lower reference limit (90%CI)	Upper reference limit (90%CI)
Ferritin (ng/ml)*	Male	60–93	983	64.18 (55.85–72.34)	865.8 (793.56–904.00)
	Female	60–87	634	52.58 (46.98–59.95)	585.88 (552.00–652.55)
DCP (mAU/ml)*	Male	60–93	2318	14.00 (14.00–14.00)	33.00 (32.00–34.00)
	Female	60–91	1725	13.00 (13.00–13.00)	29.00 (29.00–30.00)
AFP (ng/ml)^**†**^	Total	60–98	32,345	-	6.75 (6.69–6.82)
CEA (ng/ml)^**†**^	Total	60–98	32,331	-	4.85 (4.79–4.90)
CA15-3 (U/ml)^**†**^	Female	60–97	12,491	-	22.00 (21.82–22.30)
CA19-9 (U/ml)^**†**^	Total	60–98	28,707	-	28.10 (27.72–28.50)
CA125 (U/ml)^**†**^	Total	69–94	9863	-	20.96 (20.60–21.30)
CYFRA21-1 (ng/ml)^**†**^	Total	60–93	4852	-	4.66 (4.54–4.78)
NSE (ng/ml)^**†**^	Total	60–93	2886	-	19.41 (19.11–19.80)
tPSA (ng/ml)^**†**^	Male	60–98	19,454	-	5.26 (5.14–5.36)
fPSA (ng/ml)^**†**^	Male	60–98	18,259	-	1.09 (1.07–1.12)

Abbreviations: AFP, alpha-fetoprotein; CEA, carcinoembryonic antigen; CA, carbohydrate antigen; CYFRA21-1, cytokeratin 19 fragment; NSE, neuron-specific enolase; tPSA, total prostate-specific antigen; fPSA, free prostate-specific antigen; DCP, Des-γ-carboxy prothrombin; CI, confidence interval; P2.5, 2.5th percentile; P95, 95th percentile; P97.5, 97.5th percentile

*: Lower reference limit and Upper reference limit is presented as P2.5 – P97.5

**†**: Lower reference limit is not identified, Upper reference limit is presented as P95

### Verification of reference intervals

The dataset utilized for validating the corresponding RI for each biomarker exceeded 20, satisfying the fundamental criteria for RI verification. The coincidence rates of apparently healthy individuals within the calculated RI estimated by this study were uniformly above 90% (Table 4). Consequently, all calculated RIs for common tumor markers in apparently healthy elderly population of Southwestern China passed the verification. Furthermore, RIs provided by manufacturer were verified using the same data. The results indicate that the RIs provided by the manufacturer for markers such as AFP, CEA, CA15-3, CA19-9, and CA125 have also passed validation, with the concordance rates within the manufacturer RI being essentially consistent with those within the calculated RI calculated in this study. For DCP marker, the concordance rate of validation for males within calculated RI surpasses that within manufacturer RI. In contrast, the concordance rate for females remains consistent across both RIs. Detailed information can be found in Table 4.

**Table 4:** Verification of reference intervals

Analyte	Group	*N*	Calculated RI Range (Coincidence Rate, %)	Manufacturer RI Range (Coincidence Rate, %)
AFP(ng/ml)	Total	2766	0-6.75(94.86%)	0–7.00^*^ (95.55%)
CEA(ng/ml)	Total	2764	0-4.85(95.14%)	0-5.20^**†**^ (96.37%)
CA15-3(U/ml)	Female	1260	0–22.00(97.52%)	0-26.40^*^ (99.06%)
CA19-9(U/ml)	Total	2587	0-28.10(93.35%)	0–27.00^*^ (92.59%)
CA125(U/ml)	Total	1313	0-20.96(93.68%)	0–35.00^*^ (98.78%)
CYFRA21-1(ng/ml)	Total	324	0-4.66(95.37%)	0-3.30^*^ (76.23%)
NSE(ng/ml)	Total	291	0-19.41(91.07%)	0-16.30^*^ (73.19%)
tPSA(ng/ml)	Male	1485	0-5.26(91.99%)	0-4.10^**‡**^ (87.6%)
				0-4.40^**§**^(89.16%)
fPSA(ng/ml)	Male	1250	0-1.09(92.16%)	NA
Ferritin(ng/ml)	Male	72	64.18–865.80(91.67%)	NA
	Female	43	52.58-585.88(90.69%)	NA
DCP (mAU/ml)	Male	203	14.00–33.00(96.06%)	9.10–27.80^*^ (91.53%)
	Female	104	13.00–29.00(90.38%)	

Abbreviations: AFP, alpha-fetoprotein; CEA, carcinoembryonic antigen; CA, carbohydrate antigen; CYFRA21-1, cytokeratin 19 fragment; NSE, neuron-specific enolase; tPSA, total prostate-specific antigen; fPSA, free prostate-specific antigen; DCP, Des-γ-carboxy prothrombin; RI, Reference Interval

Calculated RI: Calculated Reference interval; Manufacturer RI: Reference interval provided by manufacturer

*: The reference population was not divided according to sex and age

**†**: The reference interval was established based on healthy volunteers aged 40-69years

**‡**: The reference interval was established based on healthy male volunteers aged 60-69years

**§**: The reference interval was established based on healthy male volunteers aged 70 years or older

NA: The reference intervals provided by the manufacturer do not cover the population aged 60 and above

## Discussion

The aging population is at a high risk for malignant tumors. In many countries, including China, the population is aging at an unprecedented rate, a trend expected to accelerate in the coming decades. Aging is associated with a plethora of medical issues. Therefore, personalized physical examinations and tailored test combinations to identify early warning signs in high-risk groups have become crucial in geriatric laboratory medicine. Tumor markers present in tissues, body fluids, and excreta of tumor patients, can be detected through immunological, biological, and chemical methods [23]. Noninvasive laboratory tests, such as tumor markers, play vital roles in cancer management, encompassing screening, detection, differential diagnosis, staging, treatment planning, monitoring, and recurrence detection [24]. Especially in middle-age and elderly populations, the screening of tumor markers during healthy physical examinations has important clinical value. Laboratory test results must be complemented by appropriate RIs to support clinical decision-making [25, 26]. Precise and dependable laboratory outcomes, along with suitable RIs for tumor markers, are imperative for assessing the health, tumor diagnosis, treatment, monitoring, and prognosis of elderly individuals.

The Rls are subject to variation due to factors such as ethnicity, geographical location, age, gender, and differing laboratory conditions [27]. Therefore, the CLSI EP28-A3c document advocates for each laboratory to formulate its own RIs. The guidelines recommend that the selection of appropriate reference individuals through exclusion criteria (the direct method) remains the benchmark standard. However, the direct method’s challenges—being laborious, costly, and time-intensive to amass a substantial cohort of healthy reference subjects—lead most laboratories in China to adopt the RIs supplied by reagent manufacturers. These manufacturer RIs are typically derived from data on foreign populations, casting doubt on their applicability to local demographics. The difficulty in recruiting adequate and qualified reference individuals for specialized populations means that suitable RIs are often lacking. Thus, an indirect method utilizing data-mining technology emerges as a promising alternative for establishing RIs in these groups [28–30].

During this study, we investigated the RIs for 11 common serum tumor markers, utilizing a substantial real-world data from health examinations of an elderly population in Southwestern China, compiled in the LIS of the Department of Laboratory Medicine at West China Hospital. The CLSI EP28-A3c document recommends that data sets should be “biochemically filtered” to reduce the frequency of results of participants with a higher likelihood of disease affecting the results. Therefore, according to certain inclusion and exclusion criteria, we used information related to common biochemical analytes, such as alanine aminotransferase, aspartate aminotransferase, CREA, HGB, white blood cells, and medical history, to identify and remove latent abnormalities. Exclusion of outliers strictly also provides data in the middle region of population which is closely related with healthy population. Upon analyzing data from 35,635 apparently healthy elderly individuals, we found that serum ferritin and DCP levels of the elderly population differed significantly between sexes. The RI for ferritin was notably higher in men (64.18–865.80 ng/ml) compared to women (52.58-585.88 ng/ml), which was in agreement with several literature [31]. Additionally, this study also found that the RI of serum DCP was wider in men (14.00–33.00 mAU/ml) compared to women (13.00–29.00 mAU/ml); this contrasts with the manufacturer’s reagent instructions, which suggest 9.10 to 27.80 mAU/ml, regardless of sex and age. The other indicators showed no significant differences in sex and age; therefore, we established the following overall RIs: AFP at 0 to 6.75 ng/ml; CEA at 0 to 4.85 ng/ml; CA15-3 (females only) at 0 to 22.00 U/ml; CA19-9 at 0 to 28.10 U/ml; CA125 at 0 to 20.96 U/ml; CYFRA21-1 at 0 to 4.66 ng/ml; NSE at 0 to 19.41 ng/ml; tPSA (males only) at 0 to 5.26 ng/ml; and fPSA (males only) at 0 to 1.09 ng/ml. Upon comparison with the current RIs utilized in our laboratory, notable differences were observed between the elderly and non-elderly populations. This was particularly evident in the upper limits of the RIs for CYFRA21-1, NSE and tPSA, which were significantly higher. These findings underscore the necessity for establishing age-appropriate RIs tailored specifically for the elderly demographic. All calculated RIs were validated (all coincidence rates > 90%), indicating that the new RIs for the elderly population have good clinical applicability. The CYFRA21-1, NSE and tPSA reference interval provided by the manufacturer could not be verified. Other indicators except ferritin and DCP (males) of elderly population, the coincidence rates of calculated RIs are almost equal to the RIs provided by manufacture. It shows that it is feasible and necessary to establish a specific reference interval for the old papulation by using the indirect method. Furthermore, a large-scale and systematic RI study promoted the development of combined tumor detection, which is recommended to improve the diagnostic specificity and accuracy of malignant tumors [32].

This study had some limitations. Firstly, only CA15-3 data of females were available. Therefore, the influence of sex on this indicator was not analyzed. Secondly, some indicators, such as ferritin, included relatively few reference individuals, which may have influenced the establishment of RIs. If the results of this study can be applied to other laboratories on the same platform, then it is recommended that the sample size should be expanded for further validation. It should be noted that after RIs are established and validated, they must be reviewed regularly according to the requirements of the International Organization for Standardization 15,189 document to further ensure their reliability [33].

## Conclusion

In summary, this study based on real-world data is the first to use an indirect method to establish RIs for common serum tumor markers for the elderly population in Southwestern China. It provides a basis for the screening and management of malignant tumors in the elderly population and laboratory support for the development of geriatric laboratory medicine. This study also serves as a reference model for establishing RIs for a special population that are suitable for promotion and application in clinical laboratories.

### Electronic supplementary material

Below is the link to the electronic supplementary material.

Supplementary Material 1

Supplementary Material 2

## Data Availability

No datasets were generated or analysed during the current study.

## Abbreviations

RI: Reference intervals
DCP: Des-γ-carboxy prothrombin
AFP: Alpha-fetoprotein
CEA: Carcinoembryonic antigen
CA: Carbohydrate antigen
CYFRA21-1: Cytokeratin 19 fragment
NSE: Neuron-specific enolase
tPSA: Total prostate-specific antigen
fPSA: Free prostate-specific antigen
WHO: World Health Organization
CLSI: Clinical and Laboratory Standards Institute
PRC: People’s Republic of China
