# Supplementary figures and images for: A preliminary study on the reference intervals of serum tumor marker in apparently healthy elderly population in southwestern China using real-world data
Source: BMC Cancer. 2024 May 29;24:657. doi: 10.1186/s12885-024-12408-1 (PMC11137896; doi:10.1186/s12885-024-12408-1)

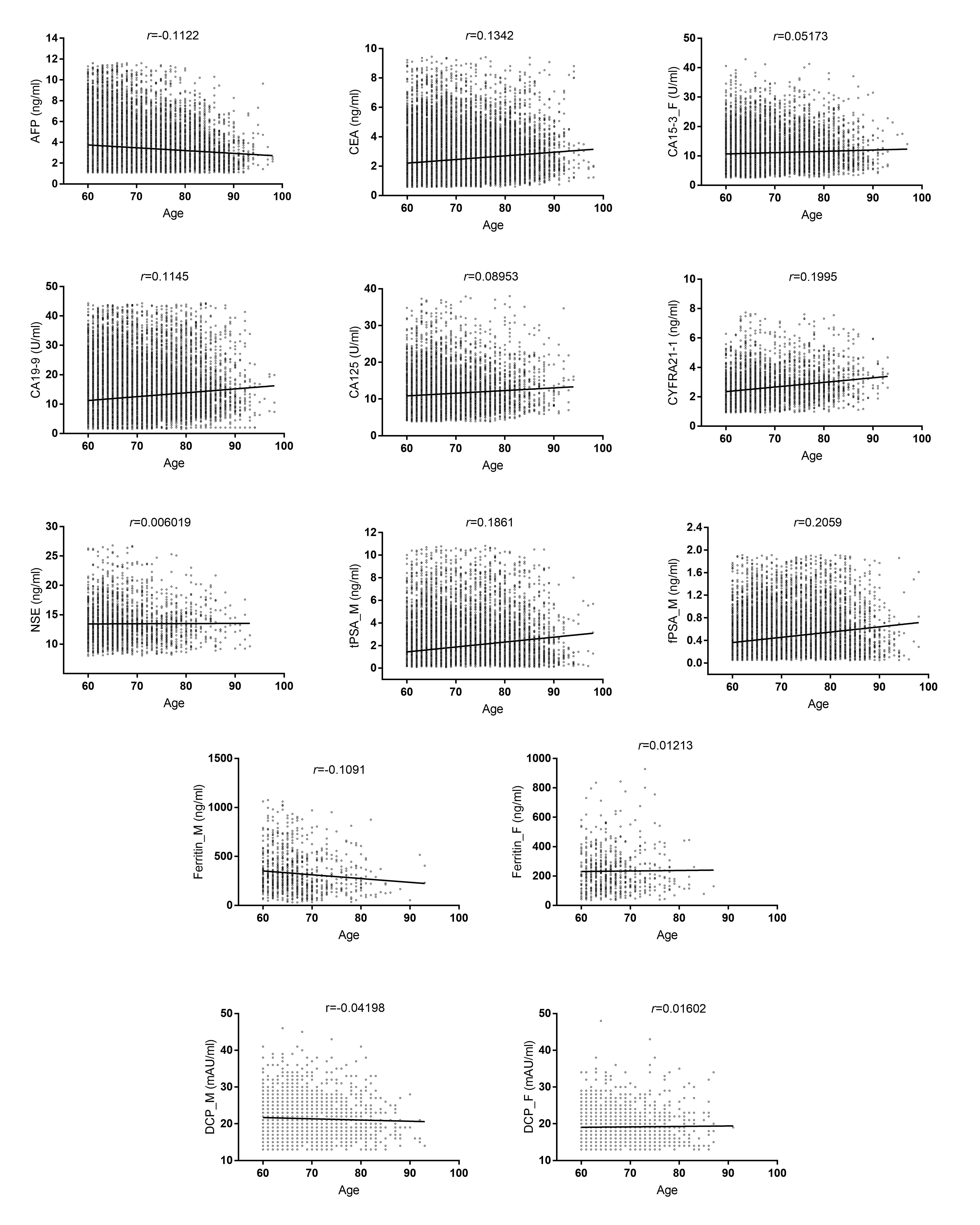

Supplement: Supplementary file 1 — Supplementary Material 1 [file 12885_2024_12408_MOESM1_ESM.png]
