# Supplementary material for: A preliminary study on the reference intervals of serum tumor marker in apparently healthy elderly population in southwestern China using real-world data
Source: BMC Cancer. 2024 May 29;24:657. doi: 10.1186/s12885-024-12408-1 (PMC11137896; doi:10.1186/s12885-024-12408-1)
Supplement: Supplementary file 2 — Supplementary Material 2 [file 12885_2024_12408_MOESM2_ESM.docx]

**A preliminary study on the reference intervals of serum tumor marker in apparently healthy elderly population in southwestern China using real-world data**

| **Table S1 The standard normal deviate test results of gender subclass** | | | | | |
| --- | --- | --- | --- | --- | --- |
| **Analyte** | **Gender** | ***N*** | **Mean+SD** | ***Z*** | ***Z**** |
| AFP（ng/ml) | male | 19494 | 3.47±1.59 | 7.613 | 34.827 |
|  | female | 12851 | 3.61±1.69 |  |  |
| CEA（ng/ml) | male | 19484 | 2.56±1.31 | 29.251 | 34.820 |
|  | female | 12847 | 2.18±1.14 |  |  |
| CA15-3（U/ml） | male | / | / | / | / |
|  | female | 12491 | 11.00±5.58 |  |  |
| CA19-9（U/ml） | Male | 17752 | 12.31±7.56 | 2.770 | 32.810 |
|  | Female | 10955 | 12.18±7.92 |  |  |
| CA125（U/ml） | Male | 5439 | 11.45±5.04 | 0.552 | 19.232 |
|  | Female | 4424 | 11.32±4.81 |  |  |
| CYFRA21-1（ng/ml) | Male | 3485 | 2.64±1.07 | 4.144 | 13.489 |
|  | Female | 1367 | 2.52±1.04 |  |  |
| NSE（ng/ml) | Male | 2018 | 13.32±3.13 | 5.022 | 10.403 |
|  | Female | 868 | 13.82±3.00 |  |  |
| tPSA（ng/ml) | Male | 19454 | 1.80±1.64 | / | / |
|  | Female | / | / |  |  |
| fPSA（ng/ml) | Male | 18259 | 0.44±0.31 | / | / |
|  | Female | / | / |  |  |
| Ferritin（ng/ml) | Male | 983 | 326.2±201.1 | 10.392^#^ | 7.787 |
|  | Female | 634 | 233.1±141.4 |  |  |
| DCP (mAU/ml) | Male | 2318 | 21.45±4.98 | 15.927^#^ | 12.313 |
|  | Female | 1725 | 19.13±4.26 |  |  |
| Abbreviation: AFP, alpha-fetoprotein; CEA, carcinoembryonic antigen; CA, carbohydrate antigen; CYFRA21-1, cytokeratin 19 fragment; NSE, neuron-specific enolase; tPSA, total prostate-specific antigen; fPSA, free prostate-specific antigen; DCP, Des-γ-carboxy prothrombin.  **#:** Z>Z*, the difference between the two groups was considered statistically significant. | | | | | |

| **Table S2 The optimal age split point calculated by decision tree method** | | | |
| --- | --- | --- | --- |
| **Analyte** | **Gender** | **Best split point (years)** | **R^2†^** |
| AFP*（ng/ml) | Total | / | / |
| CEA（ng/ml) | Total | 71 | 0.011 |
| CA15-3*（U/ml） | Total | / | / |
| CA19-9（U/ml） | Total | 74 | 0.013 |
| CA-125*（U/ml） | Total | / | / |
| CYFRA21-1（ng/ml) | Total | 71 | 0.035 |
| NSE*（ng/ml) | Total | / | / |
| tPSA（ng/ml) | Total | 70 | 0.031 |
| fPSA（ng/ml) | Total | 70 | 0.039 |
| Ferritin（ng/ml) | Female* | / | / |
|  | Male | 68 | 0.018 |
| DCP* (mAU/ml) | Female | / | / |
|  | Male | / | / |
| Abbreviation: AFP, alpha-fetoprotein; CEA, carcinoembryonic antigen; CA, carbohydrate antigen; CYFRA21-1, cytokeratin 19 fragment; NSE, neuron-specific enolase; tPSA, total prostate-specific antigen; fPSA, free prostate-specific antigen; DCP, Des-γ-carboxy prothrombin.  *：The decision tree method analysis did not recommend the best split point.  †: R^2^ is the measure of fitting degree for all subclasses after every division step in each stage. | | | |

| **Table S3 Z-test results following age group stratification by decision tree method** | | | | | | |
| --- | --- | --- | --- | --- | --- | --- |
| **Analyte** | **Age(years)** | | ***N*** | **Mean+SD** | ***Z*** | ***Z**** |
| CEA（ng/ml) | | ＜71 | 22860 | 2.31±1.23 | 20.981 | 34.820 |
|  |  | ≥71 | 9471 | 2.64±1.31 |  |  |
| CA19-9（U/ml） | | ＜74 | 22909 | 11.90±7.46 | 15.725 | 32.810 |
|  |  | ≥74 | 5798 | 13.80±8.40 |  |  |
| CYFRA21-1（ng/ml) | | ＜71 | 3418 | 2.48±0.99 | 12.367 | 13.489 |
|  |  | ≥71 | 1434 | 2.91±1.15 |  |  |
| tPSA（ng/ml) | | ＜70 | 12887 | 1.60±1.46 | 22.437 | 27.010 |
|  |  | ≥70 | 6567 | 2.20±1.90 |  |  |
| fPSA（ng/ml) | | ＜70 | 12256 | 0.40±0.27 | 23.679 | 26.568 |
|  |  | ≥70 | 6003 | 0.52±0.36 |  |  |
| Ferritin（male, ng/ml) | | ＜68 | 659 | 345.00±205.89 | 4.373 | 6.071 |
|  | ≥68 | | 324 | 287.89±185.49 |  |  |
| Abbreviation: CEA, carcinoembryonic antigen; CA, carbohydrate antigen; CYFRA21-1, cytokeratin 19 fragment; tPSA, total prostate-specific antigen; fPSA, free prostate-specific antigen. | | | | | | |

**Figure legends**

Figure S1: Scatter plot of age-dependence variation of serum tumor markers in individuals aged 60 and above. AFP, alpha-fetoprotein; CEA, carcinoembryonic antigen; CA, carbohydrate antigen; CYFRA21-1, cytokeratin 19 fragment; NSE, neuron-specific enolase; tPSA, total prostate-specific antigen; fPSA, free prostate-specific antigen; DCP, Des-γ-carboxy prothrombin; M, male; F, female.
